# Real-time monitoring of bacterial biofilms metabolic activity by a redox-reactive nanosensors array

**DOI:** 10.1186/s12951-020-00637-y

**Published:** 2020-05-24

**Authors:** Ella Yeor-Davidi, Marina Zverzhinetsky, Vadim Krivitsky, Fernando Patolsky

**Affiliations:** 1 School of Chemistry, Faculty of Exact Sciences, Tel Aviv University, 69978 Tel Aviv, Israel; 2 Department of Materials Science and Engineering, The Iby and Aladar Fleischman, Faculty of Engineering, Tel Aviv University, 69978 Tel Aviv, Israel

**Keywords:** Silicon nanowires, Biofilms, Bacteria, Nanosensors, Field effect transistors, Metabolism

## Abstract

**Background:**

Bacterial biofilms are communities of surface-associated microorganisms living in cellular clusters or micro-colonies, encapsulated in a complex matrix composed of an extracellular polymeric substance, separated by open water channels that act as a circulatory system that enable better diffusion of nutrients and easier removal of metabolic waste products. The monitoring of biofilms can provide important information on fundamental biofilm-related processes. That information can shed light on the bacterial processes and enable scientists to find ways of preventing future bacterial infections. Various approaches in use for biofilm analysis are based on microscopic, spectrochemical, electrochemical, and piezoelectrical methods. All these methods provide significant progress in understanding the bio-process related to biofilm formation and eradication, nevertheless, the development of novel approaches for the real-time monitoring of biochemical, in particular metabolic activity, of bacterial species during the formation, life and eradication of biofilms is of great potential importance.

**Results:**

Here, detection and monitoring of the metabolic activity of bacterial biofilms in high-ionic-strength solutions were enabled as a result of novel surface modification by an active redox system, composed of 9,10-dihydroxyanthracene/9,10-anthraquinone, on the oxide layer of the SiNW, yielding a chemically-gated FET array. With the use of enzymatic reactions of oxidases, metabolites can be converted to H_2_O_2_ and monitored by the nanosensors. Here, the successful detection of glucose metabolites in high-ionic-strength solutions, such as bacterial media, without pre-processing of small volume samples under different conditions and treatments, has been demonstrated. The biofilms were treated with antibiotics differing in their mechanisms of action and were compared to untreated biofilms. Further examination of biofilms under antibiotic treatment with SiNW-FET devices could shed light on the bioprocess that occurs within the biofilm. Moreover, finding proper treatment that eliminates the biofilm could be examined by the novel nanosensor as a monitoring tool.

**Conclusions:**

To summarize, the combination of redox-reactive SiNW-FET devices with micro-fluidic techniques enables the performance of rapid, automated, and real-time metabolite detection with the use of minimal sample size, noninvasively and label-free. This novel platform can be used as an extremely sensitive tool for detection and establishing medical solutions for bacterial-biofilm eradication and for finding a proper treatment to eliminate biofilm contaminations. Moreover, the sensing system can be used as a research tool for further understanding of the metabolic processes that occur within the bacterial biofilm population.

## Background

Nanostructures, such as nanowires, with unique one-dimensional morphology, are very interesting materials due to their electron-transport properties. Nanowires with predictable and controllable conductance are very important for electrical and electronic applications and serve as critical building blocks for emerging nanotechnologies [1]. Their one-dimensionality leads to an extremely high surface-to-volume ratio, which makes them great field-effect-transistor (FET) devices. Changes in the electric field at their surfaces lead to depletion or accumulation of charge carriers at their “bulk”, rendering them extremely sensitive to molecules adsorbed on their surfaces, to a point where single-molecule detection is made possible [2]. These nanostructures have led to the revolutionary concept of new devices for the detection of chemical and biological entities [3–27]. Moreover, they possess the ability to be ultra-sensitive, selective, and label-free real-time sensors.

Nonetheless, several detection limitations still need to be resolved in order to achieve the practical applicability of NW-based FET devices for sensing. One of the challenges related to sensing performance is the detection limitation due to Debye screening that occurs under conditions of high ionic strength [18]. Under these conditions, such as physiological 155 mM salt concentration, the screening length is about 1 nm, masking charge alterations occurring on surface-bound receptor molecules (i.e. proteins or DNA linkers) that are kept away from the sensor surface by a distance of 2–12 nm. Therefore, most reports regarding sensing with SiNW FETs have been carried out in solutions of low ionic strength. For optimal sensing, the Debye length must be carefully selected or samples must be diluted or desalted.

In developed countries, ninety percent of documented infections in hospitalized patients are caused by bacteria. The World Health Organization has estimated that each year, five million people die of bacterial infections, which are the second leading cause of death in the world (after cardiovascular diseases).

Bacteria often attach to surfaces and form dense aggregates called biofilms or bacterial mats. Biofilms are communities of surface-associated microorganisms living in cellular clusters or micro-colonies, encapsulated in a complex matrix composed of an extracellular polymeric substance (EPS), separated by open water channels that act as a circulatory system that enable better diffusion of nutrients and easier removal of metabolic waste products [28]. The National Institute of Health of the United States has estimated that more than 80% of the bacterial infections in the human population are biofilm-related, and that patient mortality associated with biofilms is substantial [29]. Under the protection of biofilm, microbial cells become tolerant and resistant to antibiotics and to the immune-system responses, which increases the difficulty of the clinical treatment of biofilm-based infections [30, 31]. Therefore, the effective treatment of biofilm infections with currently available antibiotics, while enabling evaluation of the treatment results, is a challenge that attracts the attention of the scientific community.

Techniques of real-time biofilm monitoring are based on a certain measured signal obtained from the biofilm under investigation. Signals such as acoustic waves, electrical fields, electric current, radiation (including light), or heat transfer can be investigated [32]. Monitoring techniques can be divided into direct measurements, related to the mass or the cell density, and indirect measurements of metabolic activity and products such as liquids or gases. Various approaches in use for biofilm analysis, from the beginning of formation to eradication, are based on microscopic, spectrochemical, electrochemical, and piezoelectrical methods [33–35]. These methods provide significant progress in understanding the bio-process related to biofilm formation and eradication. Nevertheless, the development of novel approaches for the real-time monitoring of biochemical, in particular metabolic activity, of bacterial species during the formation, life, and eradication of biofilms is of great potential importance.

Hence, biofilm monitoring by measurements of metabolic-activity products can supply information about the nature of bacterial biofilm. Strategies for non-invasive measurement of metabolic activity are diverse. For example, NMR was used decades ago to investigate substrate consumption in bacterial biofilms and to assess the effect of biofilm formation on the hydrodynamics of the surrounding liquid [36]. Bioluminescence and fluorescence methods demonstrated high sensitivity capabilities, but require labeled samples for monitoring [37–40]. The investigation and analysis of microbial metabolic activity through analysis of extracellular protein expression was demonstrated by protein-gel analysis. All the monitoring methods mentioned above are reliable and useful for biofilm research, but, on the other hand, every one of them still possesses disadvantages such as labeling, time-consuming, cost, and massive machines required for steps in the analysis, complex preparation of the detected sample or low sensitivity [41, 42]. To overcome these limitations a new technology that combines real-time detection, label-free, selective and sensitive capabilities is still required.

Cellular metabolism, and in particular glucose metabolism, has been shown to reflect the state of living cells and microorganisms. More specifically, the inhibition of bacterial metabolism of glucose consumption can be used as an index of bacterial susceptibility to drugs [43, 44]. A unique redox-reactive modification on the surface of the SiNW FET devices was previously shown to be a powerful tool for glucose sensing in high-ionic-strength solution [13, 27]. The redox system’s functional group comprising 9,10-dihydroxyanthracene (AQ), that can be oxidized or reduced in a reversible manner, which involves a significant change in the charge [13, 45], and influences the conductivity of the FET. Hydrogen peroxide (H_2_O_2_) is an oxidative species that can selectively react with the redox modification and change the level of oxidation of the system. Moreover, hydrogen peroxide has been used in a technique for real-time monitoring of metabolites by the use of corresponding oxidase enzymes to convert target metabolites to H_2_O_2_ [13]. This platform was coupled with microfluidic technologies that allowed the delivery of small volumes of detected samples on the surface of the nanosensor. Based on this work, we developed a platform that enables monitoring of the metabolic activity of biofilms based on glucose consumption, testing their susceptibility to drugs and other eradication efforts, to adjust the proper medical treatment to overcome biofilm infections. We believe that among the many different technologies available for monitoring biofilm growth, the SiNW-FET array is the most promising approach, as it affords direct, label-free, real-time, highly sensitive and specific monitoring of biofilm processes in a continuous, nondestructive manner, in their natural environment.

## Results and discussion

**Fig. 1:**
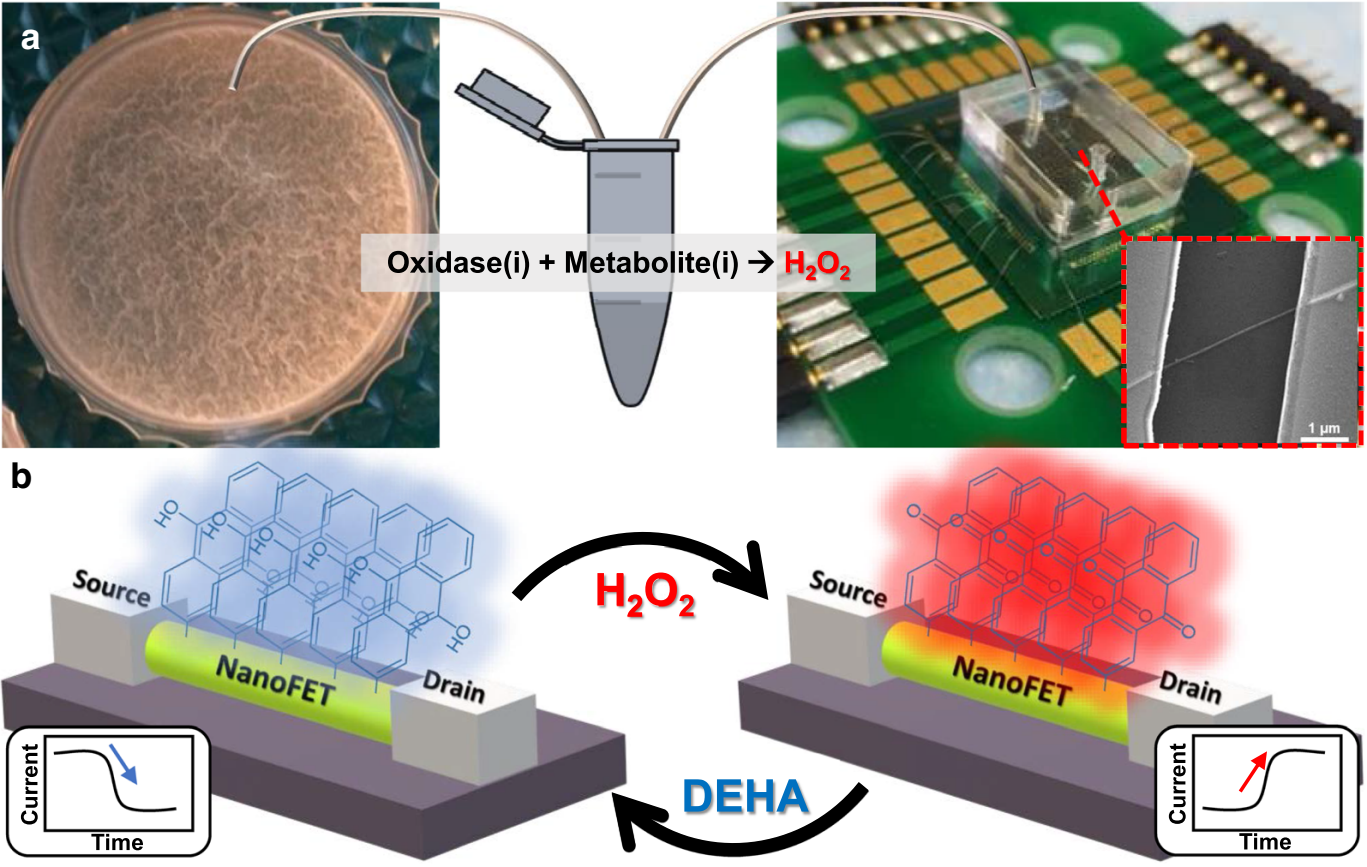
Microfluidic redox-reactive nanoFET biosensor for extracellular bacterial metabolic analysis. **a** Silicon wafer chip, with 600 nm thermal oxide layer, which contains 200 potential redox-reactive SiNW FET devices, sharing a common gate. The nanoFETs covered with PDMS microfluidic channel connected via tubing to an Eppendorf tube with a small bacterial media sample mixed with oxidase enzyme. The forming bacterial biofilms of *B. subtilis* are shown in the left panel. Inset: scanning electron microscope image of single redox-reactive nanoFET consisting of 20 nm p-type SiNW connected to the source and drain electrodes. The nanoFETs chip wire-bonded to the PCB holder, which is connected to the electrical recording system (Additional file 1: Figure S13). **b** Operation mechanism of the redox-reactive nanoFET biosensor. The redox-reactive nanoFET biosensor reversely reduced or oxidized in the present DEHA or H_2_O_2_, respectively. When the redox reactive device is oxidized, the conductivity of the device increased, and AQ moieties are formed on the nanoFET surface (right panel). On the other hand, when the redox reactive device is reduced, the conductivity of the device decreased and DHA moieties are formed on the nanoFET surface (left panel)

Based on the fact that the reaction of specific oxidase enzymes of target metabolites produce H_2_O_2_, surface modification of an active redox system composed of 9,10-dihydroxyanthracene/9,10-anthraquinone (DHA/AQ) was chosen [13]. This layer of an active redox system alternates from oxidized to reduced states and contributes charge carriers which are followed by electrical changes on the surface of the SiNW FET. Moreover, SiNW FETs modified with active redox systems enable the monitoring of metabolites without any preprocessing of the sample [13, 27], including desalting, directly from the extracellular medium of the bacterial biofilm, and offer the reuse of the nanosensor because of the unique reversible redox system. Detection and monitoring of the extracellular composition of bacterial biofilm have a considerable advantage since it is much easier to detect changes in metabolite concentrations extracellularly, as the device does not have to be in contact with the biofilm (Fig. 1a right panel). Second, the biofilm can grow in its natural environment, because the measurements are non-invasive and the device does not interfere in the ongoing biological processes (Fig. 1a left panel). Monitoring was carried out for extracellular carbon sources such as glucose, which is a highly available carbon source for bacterial growth, and the most common substrate used for studying heterotrophic metabolism [43]. By using the novel redox nano-sensor system, the influence of different treatments on *Bacillus subtilis* bacterial biofilm was monitored through glucose consumption. Here, we monitor the glucose consumption of *B. subtilis* biofilm to understand the physiological state of the biofilm. Moreover, performing sensing experiments with FET devices directly on biological samples is a challenging task because of the high ionic strength of the samples. Herein, we present a successful attempt at sensing of bacterial biofilm medium (biological samples) utilizing unique chemical modification of the FET device that manages to overcome this sensing limitation.

Surface modification of the SiNW-FET device can be utilized as a chemical gate [46]. Covalent binding of 9,10-anthraquinone-2-sulfochloride to the SiNW surface resulted in a short linkage of AQ reversible redox-reactive layer that affects the conductivity of the SiNW. When reduced with 1% v/v *N*,*N*-diethylhydroxylamine (DEHA) solution, a monolayer of DHA is formed, resulting in a decrease in conductance of the nanoFET (Fig. 1b left panel) [13]. Oxidative species, mainly H_2_O_2_, oxidizes the above redox system back to AQ monolayer on the nanoFET surface causing back the increment in the conductivity of the nanoFET device (Fig. 1b right panel). Therefore, the oxidation state can be easily reduced and a reversible sensing system of a novel redox reactive SiNW-FET device achieved. More specifically, the change in the conductivity of the device results from alterations in the population of C=O bonds [13]. The conductivity depends on the population of reduced/oxidized states of the reversible redox system moieties on the SiNW-FET surface (Fig. 1b).

The selected devices were examined for their performance in sensing buffer. Gate voltage sweep was used for transconductance measurements and the subsequent determination of the transistor regime of operation. A suitable gate voltage was selected for the performance of all the following sensing experiments. Moreover, sensing experiments were performed by monitoring the conductance of the SiNW devices over time, while target analytes were delivered to the sensing chip by the microfluidic system with the use of a syringe pump. All studies were carried out at room temperature. In addition, several control experiments were carried out to confirm that the observed conductance changes are due to the specific reaction of the modification of the redox reactive group (Additional file 1: Figure S1–S6).

**Fig. 2:**
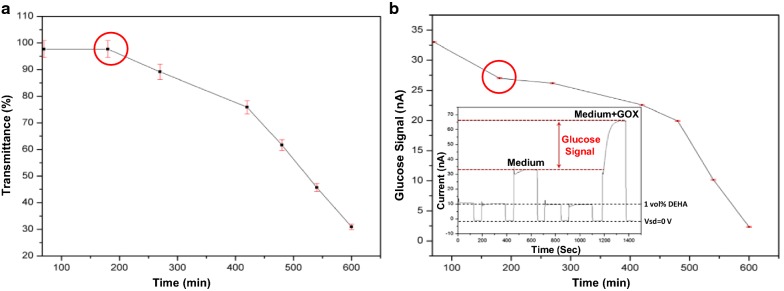
Comparison between optical and redox-reactive nanowire FET measurements of bacteria’s metabolic activity. **a** Transmittance versus time, and **b** Glucose signal versus time, as measured by spectrophotometer (at 600 nm) and by redox-reactive SiNW, respectively, during the growth of E. coli bacteria. in minimal broth medium supplemented with glucose as the only carbon source, at pH 7.3. inset of b Demonstration of the calculation method used to extract plot **b**. Before each injection of a new sample (800 µL sample, rate = 100 µL/second), the devices were switched off (Vsd = 0 V). Each measurement lasted for 180 s, during which the source-drain voltage (Vsd) was 0.3 V and the gate voltage (Vg) was 0 V. The glucose signal-versus-time curve was constructed from the current values at 180 s from the beginning of each measurement, right before the devices were switched off (Vsd = 0). The sensing conditions were chosen based on previous studies [13]. The signal was calculated by subtracting the value of the current after the addition of oxidase from the value of the current before its addition. The black dashed line marks the baseline obtained by the addition of reductant (1 vol% DEHA in medium). The error in the current values (Y-axis in **b**) is estimated to be ± 0.100 nA, the error in the transmittance values (Y-axis in **a**) is calculated as a function of the absorption measurements, as described in Additional file 1: Section 15

Monitoring bacterial metabolism is highly important for applications of food and water quality control, development of new antibiotic materials, detection of bacteria in murky solution, biotechnology, and as a research tool [47]. previous attempts to evaluate bacterial metabolic activity using the NW FET sensor was done only as pH monitoring [48]. The sensitivity and the specificity of the pH monitoring as a tool for metabolomic analysis are limited by the extracellular media buffer capabilities, which blocks the acidification of the media caused by metabolic activity, and by the variety of metabolites that can cause a change in the acidity of the medium. Therefore, we applied the analysis of *E. coli* bacterial-glucose consumption using the redox-reactive SiNW FET sensor and compared it to conventional optical methods for the analysis of bacterial metabolic activity, mainly performed by measuring the transmittance of the solution. The bacteria were grown in transparent minimal broth with glucose as the only available carbon source. In these conditions, the concentration of the glucose and the solution transmittance accordingly should decrease with time (Fig. 2a). The presented results demonstrate a high correlation between obtained glucose signals by the redox-reactive SiNW FET device to the solution’s transparency measurements. The glucose signal and the transparency of the solution have decreased with time as long as the bacterial growth continued (Fig. 2). Importantly, by the redox-reactive SiNW FET, it was possible to detect the consumption of the glucose much before any changes in the solution’s transparency were measured (marked in a red circle, Fig. 2b). As expected, the bacteria had first consumed the glucose and then used it for growth and reproduction. The fact that the redox-reactive SiNW FET has detected the bacterial metabolic activity before the optical conventional method was able to, implies the power of our approach for this task. The glucose signal was obtained by subtracting the signal of bacteria grown in broth from similar bacteria samples that were incubated for 10 min with glucose oxidase prior to redox-reactive SiNW FET measurements (Fig. 2b inset). Importantly, In Fig. 2a we demonstrate the correlation between glucose consumption and the turbidity of the bacterial culture, in order to show that our device can perform real-time monitoring of biological processes such as bacterial culturing. The robustness and characterization of redox-reactive nanodevice are shown in our previous work [13]. As can be seen in Fig. 2 inset, in order to generate the metabolite signal, we have subtracted the signals before and after GOX addition, causing the chemical background, including pH effects and the effect of other oxidative species than H_2_O_2_, to be eliminated. In addition, all the measurements were taken in a high ionic strength buffer, within a few hours, in which no significant pH changes occur. The *E. coli* is present in the medium as bacterial suspension, so its culturing and metabolic activity can be real-time monitored by simple optical means (such as turbidity, absorption, and fluorescence). In contrast, the *B. subtilis* grows in the form of a biofilm which is hardly light-permeable, making it problematic for culturing and metabolic activity monitoring by optical means. Therefore, we tested our nanodevice for real-time monitoring of *B. subtilis* biofilms.

**Fig. 3:**
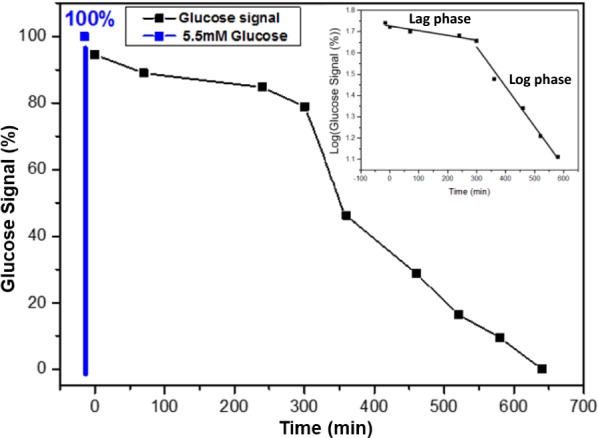
Monitoring of the metabolic activity of bacterial biofilm. The metabolic activity was monitored with the use of a redox-reactive SiNW-FET device and the time-dependence of the glucose signal is shown. First, the biofilm was grown in MSgg medium (glycerol and glutamate) and incubated at 30 °C for 40 h. Then, the MSgg medium was replaced by the glucose-based medium. The bacterial biofilm consumes all the glucose in 11 h. The minimal-broth medium contains 5.5 mM glucose at the beginning of the measurements. Inset: bacterial-growth rate curve presented as the logarithm of glucose signal versus time. The lag phase and log phase of bacterial glucose metabolism are monitored with the use of a redox-reactive SiNW FET

Next, we monitored the bacterial-biofilm metabolism which has also a critical impact on water quality control, development of new antibiotic materials, detection of bacterial biofilm in medical devices and implants, biotechnology, and as a research tool [30, 31, 49]. The following results show the metabolic activity of bacterial biofilms, obtained by measuring and analyzing the glucose consumption with the use of a redox-reactive SiNW FET. The bacteria were grown in MSgg medium to form biofilms and were then maintained with minimal-broth medium, with glucose as the only available carbon source. Under these conditions, the concentration of glucose should decrease with time as a result of consumption by the bacterial biofilm. The results show that the glucose signal decreased with time as long as the bacterial biofilm consumed the glucose within the minimal-broth medium (Fig. 3). As expected, the bacterial biofilm first consumed the glucose for maintenance of the biomass and reproduction [50]. Most importantly, it was possible to monitor the bacterial-biofilm metabolism by consumption of glucose using the redox-reactive SiNW FETs, when optical means such as turbidity are not an option.

Real-time glucose consumption by bacterial-biofilm monitored by redox-reactive SiNW FETs, for 11 h. After the biofilm was formed, the MSgg growth medium was replaced by a minimal-broth medium, with known glucose concentration. Sensing experiments begun immediately after the replacement of the medium. The results showed the glucose consumption by the film over time, beginning with a high concentration of glucose in the bacterial medium (5.5 mM) until the bacterial biofilm consumed all the glucose in the medium, and the glucose signal decreased to zero (Fig. 3). After the first 5 h of monitoring, the biofilm becomes adjusted to the new medium and the new conditions, therefore, the decrease in the glucose signal is minimal. After the biofilm has adapted to the new conditions, massive glucose consumption is observed, and, with time, the film consumes glucose until the glucose signal is zero. In addition, the logarithm of the glucose signal vs. time is calculated and the lag phase and log phase of glucose consumption is noticeable (Fig. 3 inset). The curve of log (glucose signal (%)) vs. time is similar to a theoretical bacterial-growth curve. It is important to note that the bacterial biofilm is in the maturation and sporulation stage, and the process of differentiation is not terminal at that stage. As environmental conditions change, it is possible for cells to alter their gene expression, in the case of motile or matrix-producing cells, or to germinate, in the case of spores [51]. Since the conditions of the experiment provide metabolites and nutrients, the consumption of glucose mainly refers to the maintenance of the biomass and growth of the film, which includes duplication.

**Fig. 4:**
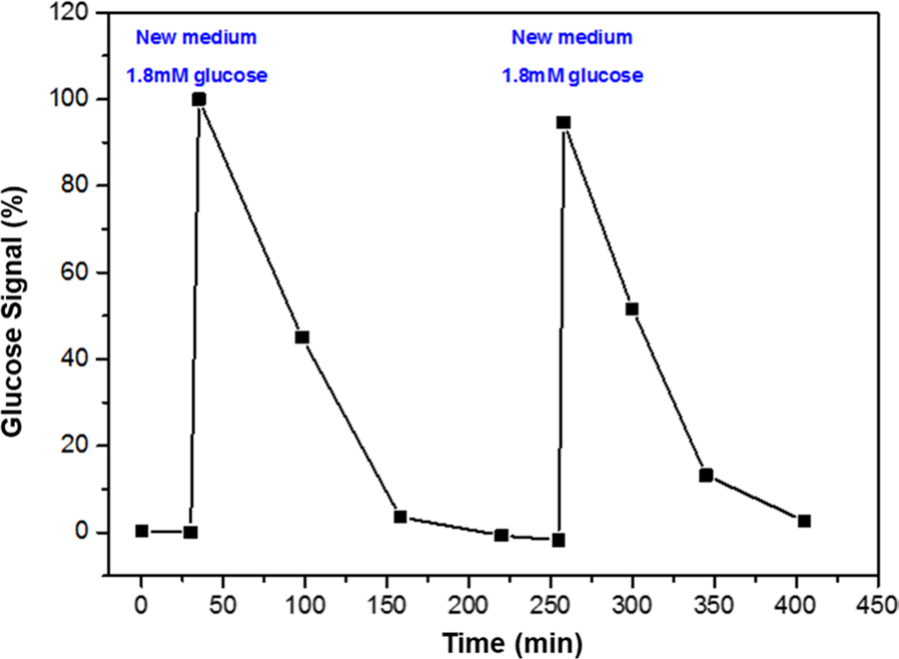
Bacterial biofilm was monitored over two cycles of glucose consumption. First, the bacterial biofilm is incubated with a minimal-broth medium containing 1.8 mM glucose, and then the medium was renewed twice. Each consumption cycle lasted approximately 150 min

The monitoring of the metabolic activity of the bacterial biofilm was performed over two cycles of glucose consumption. In the first step, the bacterial biofilm introduces to the minimal-broth medium a glucose concentration of 1.8 mM which is one-third the amount measured in the previous experiment. After the film has consumed all the glucose and the glucose signal is zero, the medium is replaced with a new one, and the monitoring continues (Fig. 4). The results show that label-free monitoring of glucose consumption can be achieved in real-time, with a SiNW FETs. In addition, reducing the concentration of glucose by a factor of three also lowers the length of the metabolic cycle. Also, the reusability and reliability of our device can be seen in Fig. 4, when we use repeatedly the same nanodevice to measure the “same real-time metabolic activity” of the same biofilm over two cycles, getting almost the same current values.

Real-time monitoring of bacterial biofilm metabolic activity is presented here, showing the metabolic response of bacterial biofilms for glucose consumption. Different conditions were examined during time, resulting in different metabolic response of the film to the variation in the carbon-source concentrations, supporting the novel qualities of the nanosensors for label-free detection in real-time, in a non-invasive and non-destructive manner.

One of the challenges in the field of bacterial biofilm infections is to find a proper treatment for the elimination of the film. It is well known that antibiotic treatment is the most effective treatment for microbial infections, but antibiotic treatments are almost impossible for the eradication of biofilm infections [30]. Moreover, to influence the biofilm by antimicrobial treatment, the concentration of the drug must be orders-of-magnitude higher than the conventional dose. The tolerance of bacterial biofilms to antibiotic treatment is well known, but their resistance mechanism is still unexplained.

**Fig. 5:**
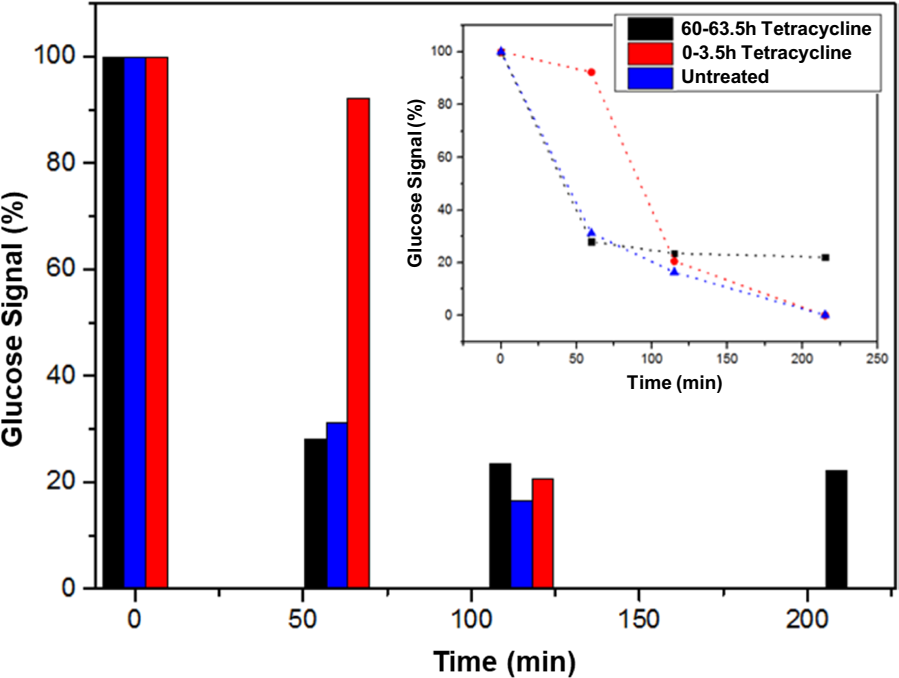
Bacterial biofilm was monitored over three cycles of glucose consumption; untreated biofilm (blue), short-term exposure to tetracycline (red), and long-term exposure (black). The long-term treatment decelerates the metabolic cycle and, in the end, ~ 20% of the glucose remains. At the beginning of each cycle, the glucose medium contains 1.8 mM glucose. Inset: trend lines of the three glucose-consumption cycles

**Fig. 6:**
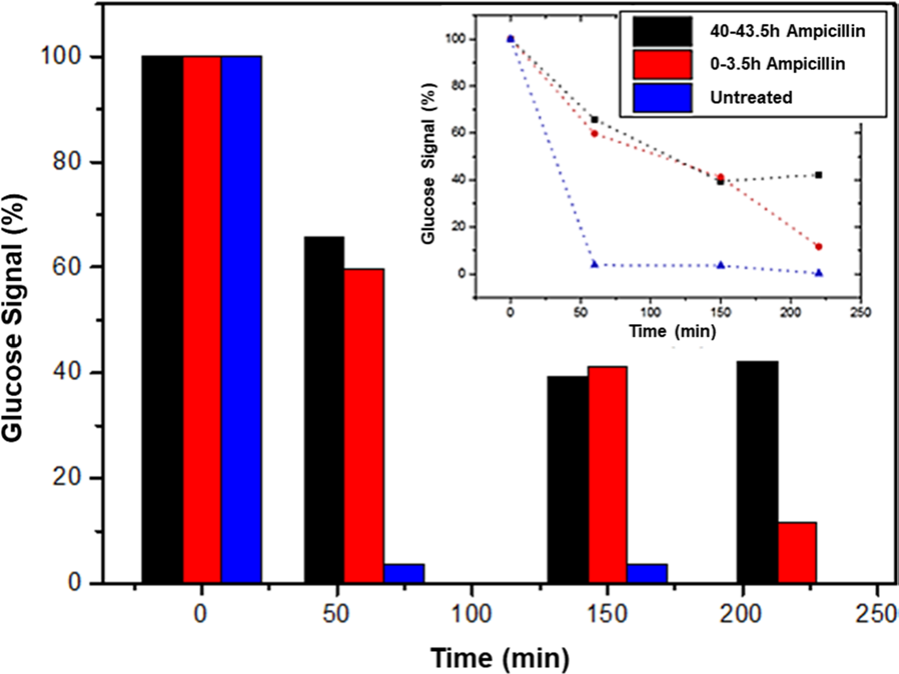
Bacterial biofilm was monitored over three cycles of glucose consumption; untreated biofilm (blue), short-term exposure to ampicillin (red), and long-term exposure (black). The long-term treatment slows the metabolic cycle and, in the end, the glucose signal shows ~ 40% remaining of the original amount. At the beginning of each cycle, the glucose medium contains 1.8 mM glucose. Inset: trend lines of the three glucose-consumption cycles

Here, we demonstrate the power of SiNW-FET nanodevices as a monitoring tool for the monitoring of the bacterial biofilm metabolic reaction in response to antibiotic treatments. The monitoring of bacterial biofilm metabolic activity reaction in response to two different antibiotic treatments is shown here (Figs. 5 and 6). Importantly, we were unable to track the bacterial viability of biofilm by optical methods such as XTT assay, due to biofilm light impermeability. Although we have witnessed a morphological change in the biofilm as a result of various treatments, we could not quantify these changes as we did with our nanodevice. In addition, the bacterial biofilm is highly resistant, even to antibiotic treatment. Therefore, we had to give antibiotic treatment for at least 40 h to see a significant difference in the metabolic activity between the control and the treated samples. The antibiotics and their concentrations were chosen according to their effect on the bacterial biofilm metabolism. The doses were calibrated to damage the biofilm metabolism without killing all of the bacteria in the biofilm.

Tetracycline is an antibiotic that inhibits bacterial growth by stopping protein synthesis. Tetracycline binds to a single site on the ribosome and blocks a key RNA interaction, which shuts off the lengthening protein chain and stops protein synthesis [52]. The *bacillus subtilis* bacteria that are treated with tetracycline stop the replication process and the formation of new cells decreases dramatically during the exponential phase of growth. Here, the metabolic activity of the bacterial biofilm to antibiotic treatment was monitored and analyzed. Bacterial biofilm was monitored for three cycles of glucose consumption; first, before antibiotic treatment with 100 µg/mL tetracycline, immediately following antibiotic treatment and during incubation with antibiotic for 220 min, and after incubation for 60 h with tetracycline. Comparing the metabolic activity of untreated biofilms to treated biofilms reveals that long-term incubation of the cells with tetracycline results in changes in their metabolic activity. The glucose signal does not reach zero, which indicates an incomplete consumption cycle. The antibiotic treatment slows the metabolic cycle, but does not eliminate the bacterial biofilm, and affects only its glucose consumption. Since the biofilm consumes the glucose, it can be related to the maintenance of the biomass of the cells that are still alive but cannot duplicate. They are protected by the extracellular matrix of the biofilm [53] and consume the glucose at some level (Fig. 5).

Ampicillin antibiotic acts as an irreversible inhibitor of the enzyme transpeptidase. It inhibits the third and final stage of bacterial cell-wall synthesis, which ultimately leads to cell lysis [54]. The results are shown for the monitoring of bacterial biofilm activity that was treated with 100 µg/mL ampicillin for different periods of time. Glucose-consumption cycles were monitored for 220 min, before antibiotic treatment, immediately after treatment with 100 µg/mL Ampicillin and after incubation with 100 µg/mL Ampicillin for 40 h. The renewal of the medium provides a source of accessible carbon and nutrient for the maintenance of the biomass. When the conditions are favorable for spores, the germination process can begin. And in the case of motile or matrix-producing cells, it is possible for cells to alter their gene expression. As a result of ampicillin treatment, a portion of bacteria cells undergoes lysis and the contents of the cells with the absorbed glucose spill back into the medium. For this reason, the glucose signals of treated biofilm decrease more slowly than the signals of the untreated metabolic cycle. The short-term antibiotic-treatment signal continues to decelerate but glucose consumption is slower than for the untreated biofilm. Long-term antibiotic treatment leads to a greater change in glucose consumption. The signal does not decrease to zero, which indicates a significant reduction of the film and damage of the bacterial cells (Fig. 6).

Three hypotheses have been formulated in an attempt to explain biofilm resistance to antibiotics. The first hypothesis that some biofilm bacteria fall into a state of slow growth due to lack of nutrients or accumulation of harmful metabolites and therefore they survive [55]. The second hypothesis is based on slow or incomplete diffusion of antibiotics into the inner layers of the biofilm. EPS matrix containing embedded biofilm bacteria represents a diffuse barrier for a great number of bacteria as a result of adsorption of antimicrobials onto cells, perhaps dead ones, in the outer parts of the biofilm [56]. The third hypothesis, up to now only a theoretical one, suggests that there is a subpopulation of cells within the biofilm whose differentiation resembles the process of spore formation. This subpopulation has a unique, highly resistant phenotype that protects them from the effects of antibiotics [55]. The results in this section support the resistance of biofilms to antibiotics; here, a high dose of antibiotics did not completely eradicate the bacterial films. The results show that the two types of antibiotics have a different effect on biofilm glucose consumption and metabolic activity, which is an outcome of the different antibiotic operating mechanism. Although none of the antibiotics completely eradicate the bacterial biofilm metabolic activity, the ampicillin had a more potent effect.

**Fig. 7:**
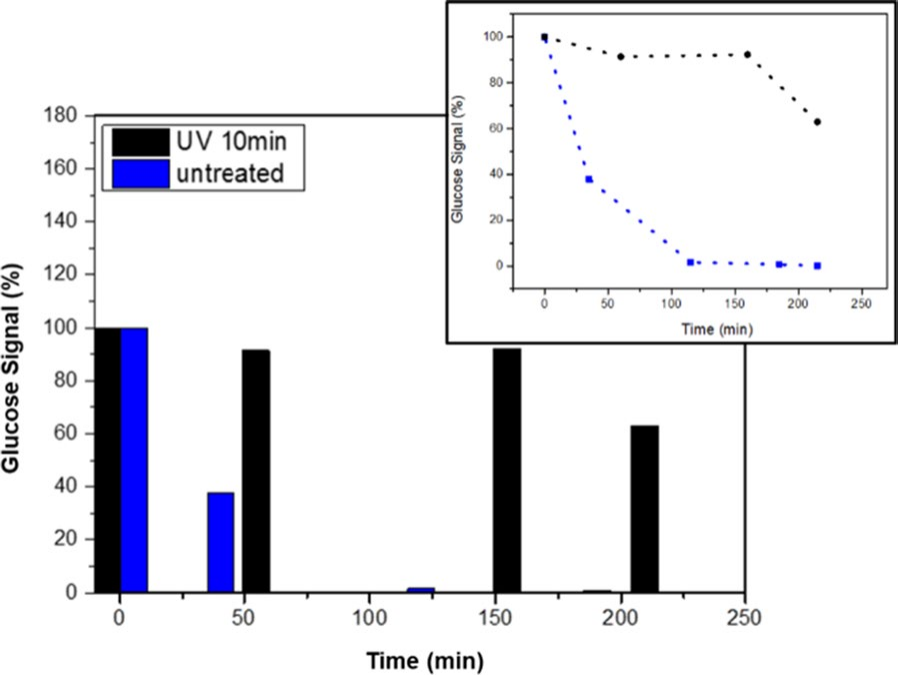
Bacterial biofilm was monitored over two cycles of glucose consumption; untreated biofilm (blue) and bacterial biofilm exposed to UV irradiation of 356 and 405 nm with intensities of 9.3 and 21.6 mW/cm^2^, respectively, for 10 min. Inset: a trend line of the two glucose-consumption cycles

Ultraviolet (UV) irradiation is germicidal and is used as a disinfection method that employs short-wavelength UV light to inactivate microorganisms by destroying their nucleic acids and disrupting their DNA, leaving them unable to perform vital cellular functions and duplication in particular. UV light breaks molecular bonds within the bacterial cell DNA, breaking thymine into dimers that damage the cell-life function [57, 58]. Moreover, it has been demonstrated that 405 nm light inactivates different gram-positive bacteria at an irradiance of 10 mW/cm^2^ [59]. Here, bacterial biofilm was exposed to 356 and 405 nm light irradiation for 10 min at 9.3 and 21.6 10 mW/cm^2^, respectively, and the metabolic activity was monitored for 220 min. The results reveal that after 160 min of irradiation, the bacterial biofilm barely consumed glucose. At some point, it began to consume glucose, probably following the recovery of the film from the UV shock, as observed by the decrease in the glucose signal **(**Fig. 7).

Aggressive treatment by UV irradiation resulted in inhibition of glucose consumption, supporting the fact that microorganisms can be damaged by UV light. Because of the remarkable capabilities of the biofilm to survive different attempts of eradication [30], it continues to consume glucose, which indicates life activity.

## Conclusions

Real-time detection of biofilm metabolic activity is an active field of research. It is driven by the need to understand and control the growth of biofilms in a number of applications, including clinical devices, industrial facilities, natural environments, and basic research. Despite significant progress in the ability to monitor the growth of biofilms, and related living cells, the sensitivity, and selectivity of such sensors are still limiting factors. We believe that among the many different technologies available for monitoring biofilm growth, the SiNW-FET array is the most promising, as it affords a real-time, direct, label-free, high sensitive and specific means for monitoring biofilm processes in a continuous, nondestructive manner.

Here, detection and monitoring of the metabolic activity of bacterial biofilms in high-ionic-strength solutions were enabled as a result of a novel surface modification by an active redox system, composed of 9,10-dihydroxyanthracene/9,10-anthraquinone, on the oxide layer of the SiNW, yielding a chemically-gated FET array. With the use of enzymatic reactions of oxidases, metabolites can be converted to H_2_O_2_ and monitored by the nanosensors. Here, the successful detection of glucose metabolites in high-ionic-strength solutions, such as bacterial media, without pre-processing of small volume samples under different conditions and treatments, has been demonstrated.

In this study, we have demonstrated the monitoring of bacterial-biofilm glucose consumption using a nanosensor based on a SiNW-FET array. A metabolic-activity curve was constructed and analyzed. Previous studies have shown that biofilm is polymorphic and can adjust to changes in the amount of nutrients. This was demonstrated by experiments with different glucose concentrations. When the glucose concentration is high, microcolonies grow rapidly. When the concentration decreases, the biofilm biomass is reduced [60]. Here, different glucose-concentration media exhibited different consumption cycles of metabolite. As the glucose concentration is reduced, the metabolic cycle is shorter. Moreover, the monitoring of more than one glucose-consumption cycle on the same bacterial biofilm was successfully demonstrated, showing that the sensing system does not harm or interfere with the biosamples.

The biofilms were treated with antibiotics differing in their mechanisms of action and were compared to untreated biofilms. As a result of the different mechanisms of action of tetracycline and ampicillin, the reaction of the bacterial biofilm was different in each case. Treatment with ampicillin inhibits glucose consumption from the beginning of the treatment; a short period of treatment does not harm the biomass to a level that glucose consumption stops. Long-term incubation with ampicillin leads to partial lysis of the biomass, and therefore only 60% of the glucose is consumed. Because of their resistance mechanism, the antibiotic treatments do not fully eradicate and eliminate the bacterial biofilm. In the case of tetracycline, short-term treatment begins with slow glucose consumption increasing to full consumption of the metabolite; long-term incubation with tetracycline damages the biofilm and causes changes in the metabolic activity, but does not lead to a complete elimination of the bacteria. There have been attempts to explain the resistance of biofilm to antibiotics, and a hypothesis has been formulated to explain the mechanism of this resistance [55]. Further examination of biofilms under antibiotic treatment with SiNW-FET devices could shed light on the bioprocess that occurs within the biofilm. Moreover, finding proper treatment that eliminates the biofilm could be examined by the novel nanosensor as a monitoring tool.

In addition, aggressive UV irradiation treatment was performed and was followed by inhibition of glucose consumption. Bacterial biofilm was damaged, but after more than three hours, the bacterial species recovered, consumption of glucose was renewed, and the lowering of the glucose signal was recorded. For untreated biofilms, all experiments gave comparable results. Those treatment curves, reflecting metabolic responses to non-invasive biofilm-eradication attempts in real-time, differed from those obtained with the established methods since they are label-free, selective, and highly sensitive.

Our unique sensing platform of SiNW-FET devices, modified with a reactive redox system, enables the detection of metabolites on the basis of an enzymatic reaction. Since enzymes are designed by natural selection to specifically and selectively fit their biochemical role [61] and importantly, because enzymes are highly efficient catalysts, the required amount of enzymes for the reaction is minuscule. Therefore, the sensing system coupled to an enzymatic reaction has a significant advantage of selectivity over non-enzymatic methods.

To summarize, the combination of redox-reactive SiNW-FET devices with micro-fluidic techniques enables the performance of rapid, automated, and real-time metabolite detection with the use of minimal sample size, noninvasively and label-free. This novel platform can be used as an extremely sensitive tool for detection and establishing medical solutions for bacterial-biofilm eradication and for finding a proper treatment to eliminate biofilm contaminations. Moreover, the sensing system can be used as a research tool for further understanding of the metabolic processes that occur within the bacterial biofilm population. Further technological development, together with advanced research, suggests that the system can be integrated into medical implants for monitoring bacterial activity in order to prevent future infections.

## Supplementary information

**Additional file 1: **(1) Characterization and calibration of the sensing system; (2) Synthesis of p-type silicon nanowires via chemical vapor deposition; (3) Fabrication of silicon nanowire field-effect-transistor array; (4) Electrical characterization of SiNW devices with the use of a water gate; (5) Scanning electron microscope analysis; (6) Surface modification; (7) Preparation of 9,10-anthraquinone-2-sulfochloride; (8) Surface modification of SiNW-FET array with 9,10-anthraquinone-2 sulfochloride; (9) Fabrication of the microfluidic channel as a delivery system; (10) Electrical measurements system of SiNW-FET devices; (11) *E. coli* culture handling; (12) Formation and maintenance of bacterial biofilms; (13) Measurement protocol; (14) Enzymes, cofactors, antibiotics, and metabolites used for metabolic analysis; (15) Error analysis.

## Data Availability

All data generated or analyzed during this study are included in this published article.

## Abbreviations

EPS: Extracellular polymeric substance
FET: Field-effect-transistor
AQ: 9,10-Dihydroxyanthracene
DHA: 9,10-Dihydroxyanthracene
H_2_O_2_: Hydrogen peroxide
DEHA: *N*,*N*-diethylhydroxylamine
UV: Ultraviolet
