# Supplementary material for: Real-time monitoring of bacterial biofilms metabolic activity by a redox-reactive nanosensors array
Source: J Nanobiotechnology. 2020 May 24;18:81. doi: 10.1186/s12951-020-00637-y (PMC7247256; doi:10.1186/s12951-020-00637-y)
Supplement: Supplementary file 1 — Additional file 1: (1) Characterization and calibration of the sensing system; (2) Synthesis of p-type silicon nanowires via chemical vapor deposition; (3) Fabrication of silicon nanowire field-effect-transistor array; (4) Electrical characterization of SiNW devices with the use of a water gate; (5) Scanning electron microscope analysis; (6) Surface modification; (7) Preparation of 9,10-anthraquinone-2-sulfochloride; (8) Surface modification of SiNW-FET array with 9,10-anthraquinone-2 sulfochloride; (9) Fabrication of the microfluidic channel as a delivery system; (10) Electrical measurements system of SiNW-FET devices; (11) E. coli culture handling; (12) Formation and maintenance of bacterial biofilms; (13) Measurement protocol; (14) Enzymes, cofactors, antibiotics, and metabolites used for metabolic analysis; (15) Error analysis. [file 12951_2020_637_MOESM1_ESM.docx]

**Additional file**

Real-time Monitoring of Bacterial Biofilms Metabolic Activity by a Redox-Reactive Nanosensors Array

Ella Yeor Davidi^1^, Marina Zverzhinetsky^1^, Vadim Krivitsky^1*^, and Fernando Patolsky^1,2*^

1. School of Chemistry, Faculty of Exact Sciences, Tel Aviv University, Tel Aviv, 69978, Israel.

2. Department of Materials Science and Engineering, the Iby and Aladar Fleischman Faculty of Engineering, Tel Aviv University, Tel Aviv 69978, Israel.

**1. Characterization and Calibration of the Sensing System**

Characterization and calibration of the sensing system were performed by activation of the devices and recording current changes related to the chemical reaction on the surface of the NWs. Current changes were accomplished by delivering specific chemical solutions that activate the redox system, and by applying a source-drain voltage (Vsd) of 0.3 V. The recorded current signals were analyzed by utilizing the ratio between the signals of the reduction and oxidation states of the system since the change in the conductivity depends on the population ratio of reduced/oxidized states. This indicates the concentration of the peroxide in the detected samples. First, an examination of the reduced and oxidized states of the redox-reactive SiNW-FET was performed electrically by complete reduction and oxidation of the modified surface to enable the characterization of the signals. Reduction of the 9,10-anthraquinone groups on the SiNW by a reducing agent - 1% v/v DEHA in PBS - and oxidation with 10mM $H_{2}O_{2}$. When the SiNW device was exposed to 1% v/v DEHA in PBS solution (physiological conditions with high ionic strength), the 9,10-anthraquinone was reduced and the conductivity of the nano-device decreased as a result. On the other hand, a solution of 10mM $H_{2}O_{2}$ in PBS oxidized the 9,10-dihydroxyanthracene moieties on the SiNW surface, resulting in increased conductivity of the device, which signifies a successful oxidation process of the redox-reactive system on the surface of the SiNW FET device **(Figure S1a)**.


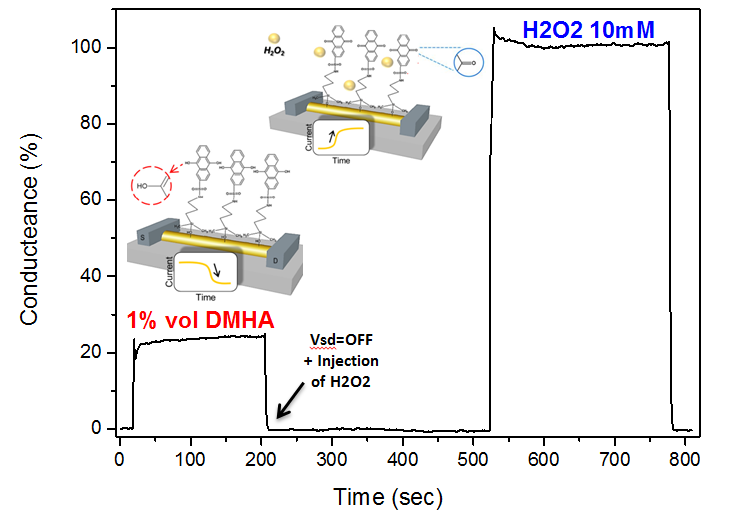


**10 mM H_2_O_2_**

**1 %v/v DEHA**

**b**

Calibrated Response (%)

**a**


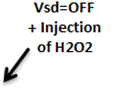

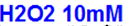

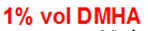

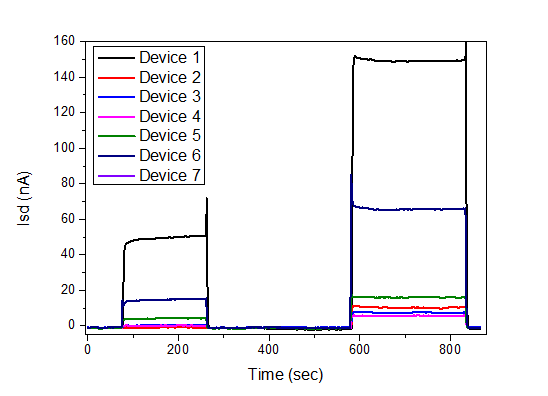


**1 %v/v DEHA**

**10 mM H_2_O_2_**

Current (nA)

**Figure S2. a**. Signal of the two oxidation states of 9,10-anthraquinone on the surface of the NW. As a response to the chemical reaction that occurs on the surface, there are current changes, increases of the signal in the oxidized state and lowering of the current in the reduced state. Measurements were performed at pH 7.4 in 150mM phosphate-buffered saline and 700μl of the sample were injected. Before sample injection, the devices were turned off (Vsd=0 volt). During the measurement, the source-drain voltage (Vsd) was 0.3V and the gate voltage (Vg) was 0V. **b**. Showing consistency of the current conductance within the SiNWs from seven different devices on the same sensor chip, modified with a redox-active layer. Under the chemical gate, the electrical signal varies because of different solutions that reduce and oxidize the system.


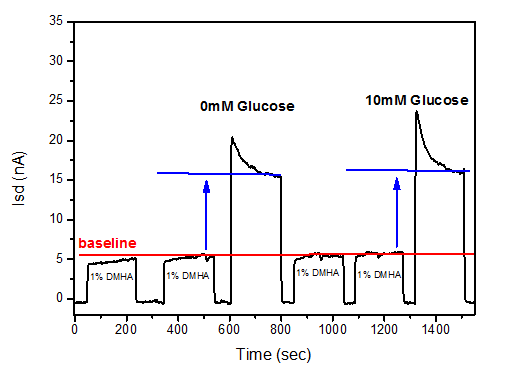
The fabricated devices in the sensor chip show variation in performance in regards to device properties such as conductance, threshold voltage, and transconductance[1]. On the other hand, most of the FET devices on a single sensor chip show consistency. The consistency between different nanodevices, on the same sensor chip, during the same time period, was examined by performing sensing of $H_{2}O_{2}$ after reduction of the sensing system with 1% v/v DEHA solution **(Figure S2b)**. As a control, SiNWs were tested in minimal broth solutions (high-ionic-strength solutions) containing different concentrations of glucose, to prove the specificity of the devices solely for $H_{2}O_{2}$. Since the modification is specific to $H_{2}O_{2}$, changes in glucose concentration in the tested solutions will yield no difference between the currents measured from the nanodevices **(Figure S3)**.


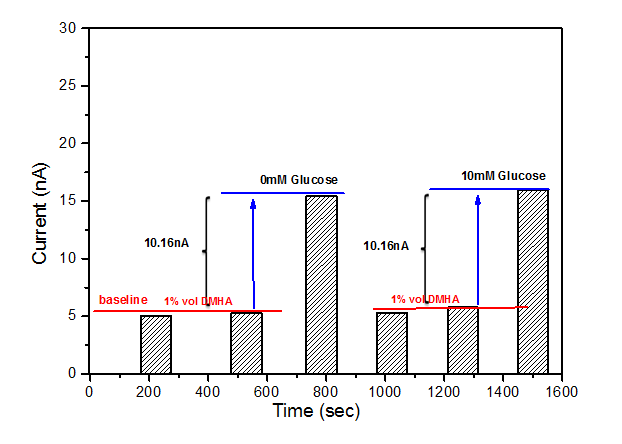


**Baseline - 1 %v/v DEHA**

**Baseline - 1 %v/v DEHA**

**Current (nA)**

**Time (Sec)**

**Current (nA)**

**Baseline - 1 %v/v DEHA**

**Figure S3**. Sensing currents for different concentrations of glucose in minimal broth medium. Histogram of current values at 180 seconds from the beginning of each measurement. Signals of minimal broth medium with no glucose (0mM) and with 10mM of glucose were analyzed by subtraction of the baseline values from the current values. The signals were equal with a value of 10.16nA. **Inset**: raw data of the sensing experiment. The surface was reduced by the addition of 1% v/v DEHA in phosphate-buffered saline (150mM, pH=7.45). Before injecting a new sample (700μl sample, rate=100μl/second), the devices were turned off (Vsd=0 V). Each measurement lasted for 180 seconds, during which the source-drain voltage (Vsd) was 0.3V and the gate voltage (Vg) was 0V.

Characterization of the nanodevices demonstrate that the FETs modified with redox-reactive systems are specific to $H_{2}O_{2}$. Solutions with different glucose concentrations yielded similar signals, which proves that different chemical environments do not affect conductivity. Moreover, different devices on a single sensor chip show consistency.

The redox-reactive SiNW FET presented here was designed to perform in physiological solutions, such as cell culture. Target metabolites can be converted to $H_{2}O_{2}$ with the use of the corresponding oxidase enzymes[2, 3]. Here, glucose oxidase was used as the oxidase enzyme to release $H_{2}O_{2}$ at the end of the reaction with glucose in the bacterial medium as follows:

$$D-Glucose+H_{2}O+O_{2} \underset{\to}{\mathrm{GOX}} D-Gluconic Acid+H_{2}O_{2}$$

The hydrogen-peroxide product oxidizes the active layer on the surface of the NW, and charge-carrier-accumulation occurs, which results in an increase in the conductance through the nanodevice. Most significantly, the reduction of the nanodevice surface by the 1% v/v DEHA solution enables a return of the system to the baseline conditions which indicates reversible and repeatable capabilities of our redox-reactive SiNW FET. First, a calibration curve was obtained, which corresponds to the activation of the enzyme in the detected samples at different concentrations of glucose in the minimal-broth medium **(Figure S4a)**.

**a**

a)

a)


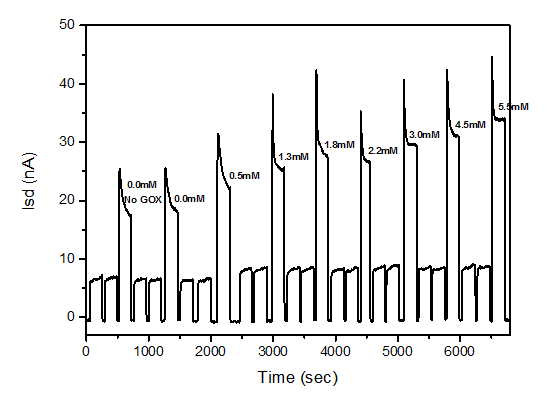


**Current (nA)**


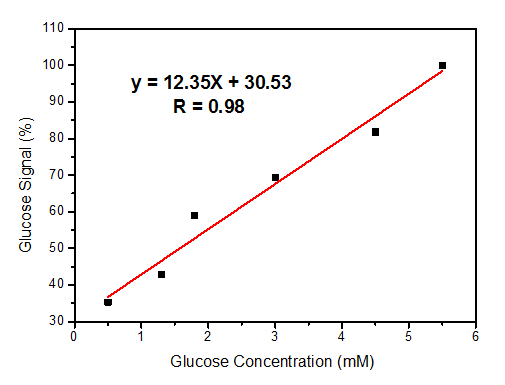


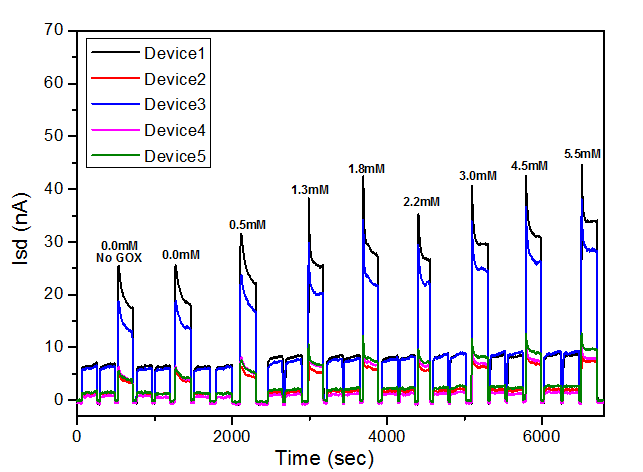


**c**

**b**

**Current (nA)**

**Glucose Signal (%)**

**Time (Sec)**

**Glucose Concentration (mM)**

**Figure S4.** Sensing of glucose with the use of GOX in physiological solution by a redox-reactive SiNW FET: **a)** Sensing of $H_{2}O_{2}$ in the minimal-broth medium after activation of the sample by GOX. The sensor surface was first reduced by 1% v/v DEHA in phosphate-buffered saline (150mM, pH=7.45). Before injecting a fresh sample (700μl at 100μl/second), the devices were turned off (Vsd=0V). Each measurement lasted for 180 seconds, during which the source-drain voltage (Vsd) was 0.3V and the gate voltage (Vg) was 0V. **b)** The calibration curve represents the correlation between the glucose signal and the concentration of glucose in the medium. The solid line represents the fit on the assumption of linear correlation, which yielded a correlation coefficient of ∼0.98. The 100% reading is the glucose signal at 5.5mM glucose in the minimal-broth medium. The glucose signal was calculated by subtraction of the baseline value from the current value at 180 seconds. **c)** Current recorded from all functional devices show consistency.


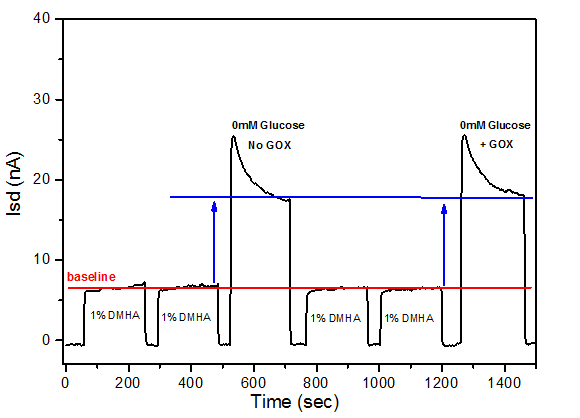
It is important to emphasize that when minimal-broth medium samples without glucose and with glucose oxidase were introduced separately to the device, there was no difference between the two signals (**Figure S5)**. Also, sensing experiments of constant glucose concentration of 1.8mM in the minimal-broth medium was performed. The medium was tested without bacteria and resulted in repeatable measurements. As expected, for a fixed concentration of glucose in the samples, similar glucose signals were recorded. The average glucose signal was 23.91nA, with a standard deviation of 0.515nA and a standard-deviation error of 2.15%.

**Baseline - 1 %v/v DEHA**

**Time (Sec)**

**Current (nA)**

**Figure S5.** Control experiment: sensing of minimal-broth medium without glucose, and with and without the GOX enzyme, showing no difference. The sensor surface was first reduced by 1% v/v DEHA in 150mM PBS (pH=7.45). The sample volume was 700μl and it was injected at a rate of 100μl/second. Before injecting a fresh sample (700μl sample, rate=100μl/second), the device was turned off (Vsd=0V). Each measurement lasted for 180 seconds, during which the source-drain voltage (Vsd) was 0.3V and the gate voltage (Vg) was 0V.

Real-time curves for the calibration of the sensitivity of the nanodevices are presented here for the whole range of glucose concentrations. The samples were injected sequentially, each injection followed by 1% v/v DEHA, showing that the signal returns to the baseline. Specific device response to the enzymatic reaction is measured and characterization of the SiNW FETs is enabled.

The glucose signal was calculated by subtracting the signal of the bacterial biofilm medium from that of the same solution following 10 minutes of incubation with glucose oxidase. The bacterial biofilm medium may contain peroxide and other secretions that can interact with the redox-reactive system; therefore, setting the "blank" of the sample in every sensing experiment is required to obtain only the signal of the $H_{2}O_{2}$ that resulted from the reaction of GOX with glucose in the medium. Three signals were obtained. The first signal was that for DEHA alone, which served as the baseline. The second was that for biofilm medium only and the third signal resulted from biofilm medium and GOX. The glucose signal, in nA, is the difference between the second and third signals **(Figure 5)**.


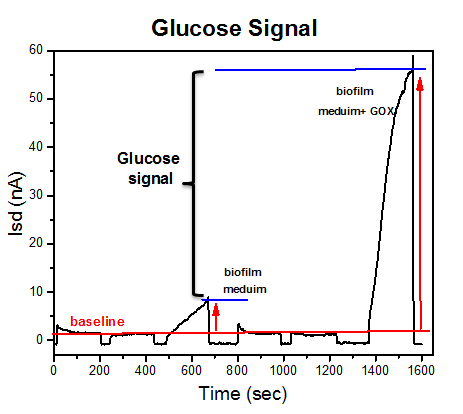


**biofilm medium+GOX**

**biofilm medium**

**Figure S6.** Raw data of one glucose signal measurement. The current is recorded as a function of time and the glucose signal is indicated. The order of injections: 1% v/v DEHA in 150mM PBS pH=7.45 X2, biofilm medium, 1% v/v DEHA X2 and biofilm medium+GOX that was incubated for 10 minutes. Before injecting a fresh sample (700μl sample, rate=100μl/second), the devices were turned off (Vsd=0V). Each measurement lasted for 180 seconds, during which the source-drain voltage (Vsd) was 0.3V and the gate voltage (Vg) was 0V.

**2. Synthesis of p-Type Silicon Nanowires via Chemical Vapor Deposition (CVD)**

SiNWs were synthesized by CVD via the VLS mechanism as follows: As catalyst sites for the VLS-CVD growth of silicon nanowires, 20nm gold nanoparticles (Ted Pella) were used. Initially, a poly-L-lysine (PLL) solution (Ted Pella) was applied to a bare Si(100) growth substrate to promote the adhesion of the gold nanoparticles to the silicon substrate as an electrostatic binding agent. Then, 20nm gold nanoparticles were deposited on the silicon wafer, organic traces of PLL were removed by a 100W oxygen-plasma step for five minutes. The silicon wafer with the gold nanoparticles was placed in a quartz furnace. To provide a p-type dopant SiNW, high-purity silane (SiH_4_) and diborane (B_2_H_6_, 100 ppm in H_2_ balance gas) with a B: Si ratio of 1:4000 were used as reactants during the growth.

**3. Fabrication of Silicon Nanowire Field-Effect-Transistor Array**

The guideline of this study is the use of SiNW-based devices for bio-applications; therefore SiNW-FET-array chips were fabricated as shown in a flow chart in **Figure S6**. For every chip, the fabrication process was performed under the same conditions and with the same equipment. The fabrication protocol[4] was the basis of the process and some alterations were performed. SiNW-FET devices were fabricated by photolithography on highly-doped silicon wafers (1,0,0) with 600nm thermal oxide (Silicon Quest International). First, outer electrodes were defined by photolithography followed by a multilayer photoresist structure consisting of 500nm LOR5A (Microchem) and 500nm Shipley 1805 (Shipley) the development performed in an AZ726 developer. After exposure metal contacts were evaporated by thermal evaporation of Cr/Au (5/60 nm, respectively). SiNWs were then deposited by random dispersion by pipetting multiple 0.2μl drops of suspended NWs in ethanol. Next, source and drain electrodes were determined by photolithography as described above, the chip was dipped in a 6:1 BOE (buffered oxide etchant, NH_4_F/HF) solution for five seconds and immediately metalized by thermal evaporation of Ni (70nm). Following this, a passivation step of Si_3_N_4_ (100nm-thick) insulating layer deposited by plasma-enhanced chemical vapor deposition (PECVD) and a layer of 10nm alumina coating performed by atomic layer deposition (ALD, Savannah 200 system, Cambridge Nanotech). The chip was then subjected to lift-off in NMP (N-Methyl-2-pyrrolidone) and the fabrication was concluded by an annealing process at 380°C for two minutes in forming gas (10% H_2_/ 90% N_2_) by a rapid thermal processor (RTP).


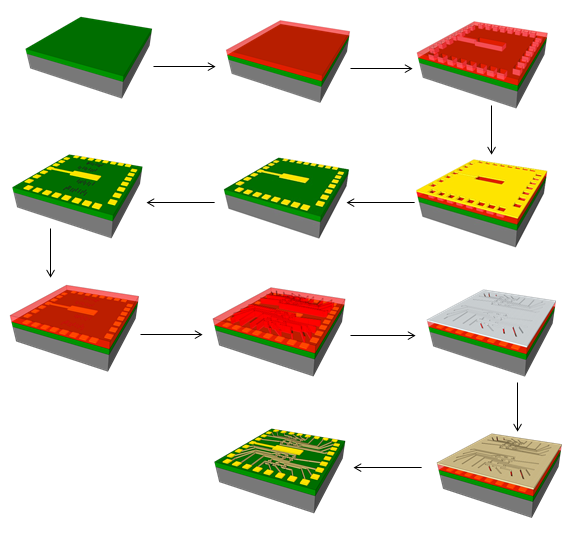


HF etching of exposed NWs reigns

+ Ni evaporation

Photolithography

of source and drain

electrodes

Photolithography of outer contact pads

20nm SiNWs deposition on sabstreat

Photoresist lift off with NMP

Photoresist lift off with NMP

Deposition of SiNx +

Alumina coating passivation

Cr + Au evaporation

LOR5A + S1805

LOR5A + S1805

**Figure S7**. Flow chart for sensor-chip fabrication.

**4. Electrical Characterization of SiNW Devices with the Use of a Water Gate**

Characterization of the basic electrical properties of the SiNW devices before surface modification is crucial for the understanding of the quality of the SiNW FET. Quality control and characterization were performed by the probe station in deionized water (DIW) used as a water-gate. As a result of the random deposition of the SiNWs, between source and drain terminals we can find either NW or no NW and in some cases a cluster of NWs. Therefore, out of 200 potential devices on a single chip, only about 20% are found functional. The electrical properties in cases where a wire is present are analyzed to understand if the device is quality p-type FET or may suffer from bad contacts, breakage or cluster of wires, etc. Every device was electrically measured by source-voltage sweep, which enabled the selection of the source-drain voltage (V_sd_) that allowed full-scale sensitivity. The device transconductance (TC) performance was determined by scanning the gate voltage (V_g_) over fixed source voltage, as well as threshold voltage (V_th_) and carrier mobility (μ). Devices with the best performance were selected for future sensing applications and a map of the sensor chip was drawn with the active FETs.


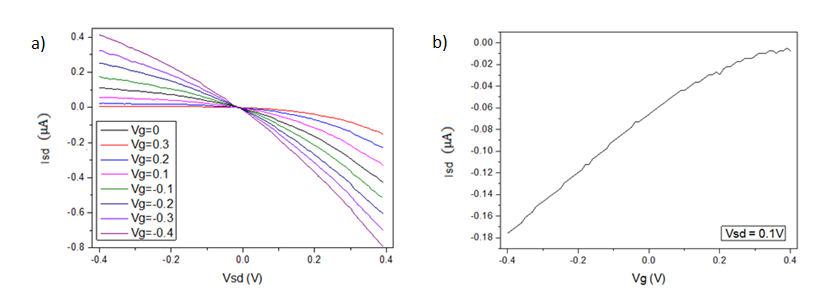


**b**

**a**

**Figure S8.** **a.** Current of source-drain (I_sd_) versus source-drain voltage (V_sd_) behavior and applied gate voltages produced predictable changes in the I-V curve for a typical p-type SiNW FET. **b.** I_sd_ versus V_g_ recorded for the same p-type SiNW-FET device at a source-drain voltage of 0.1V. These results indicate that the prepared devices indeed act as p-type FETs, showing a gate-dependent behavior and demonstrating ohmic behavior of nanowire devices.

**5. Scanning Electron Microscope Analysis**

Scanning Electron Microscope (SEM) enables imaging of the fabricated devices with high resolution. Therefore, the quality of the SiNW devices was analyzed for a single-device level with the use of a Quanta 200 FEG environmental-scanning electron microscope (ESEM) **Figure S7**. Estimating the contacts of the source and drain electrodes with the nanowire and understanding the assembly of the FET provide complementary information about the device in addition to its electrical properties.


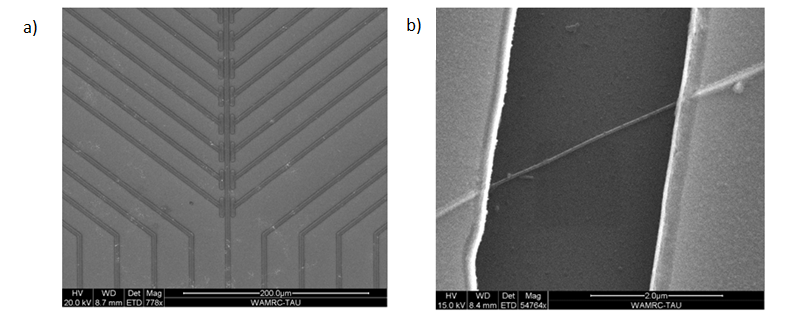


**b**

**a**

**Figure S9.** **a.** SEM image of one row of addressable device elements from a sensor chip. **b.** High-resolution SEM image of a good silicon nanowire device, the SiNW connected between the source and drain electrodes.

**6. Surface Modification**

A nanosensor based on SiNW FETs requires surface modification to be chemically gated. For that purpose, following fabrication, the NWs were covalently linked by specific agents to perform monitoring of bacterial-biofilm metabolic activity. Since SiNWs have an oxide layer on their surface, and that the chemistry of silicon oxide is well known[5], a covalently bonded layer of 3-aminopropyldimethyl-ethoxysilane (APDMES), followed by the linkage of 9,10-anthraquinone-2-sulfochloride constitute an active sensing layer. APDEMS link to the oxide surface results in a surface terminating of amine groups that can be bonded to sulfochloride groups. Therefore, sodium 9,10-anthraquinone-2-sulfonate, commercially purchased from "Sigma- Aldrich", needs to be converted into 9,10-anthraquinone-2-sulfochloride.

**7. Preparation of 9,10-anthraquinone-2-sulfochloride**

The preparation of 9,10-anthraquinone-2-sulfochloride began with commercial-grade sodium 9,10-anthraquinone-2-sulfonate (743038, Sigma-Aldrich) mixed with oxalyl chloride (O880, Sigma-Aldrich), N,N-dimethylformamide (227056, Sigma-Aldrich) in toluene (244511, Sigma-Aldrich). In the first step, 5g/0.0158mol of sodium anthraquinone-2-sulfonate and 150ml of toluene were mixed in a 250ml round-bottomed flask, heated under reflux for two hours at 110ᵒC until the mixture was dry and cooled to 60ᵒC. 6ml of oxalyl chloride and two drops of N,N-dimethylformamide (~100μl) were added to the flask which was heated under reflux for eight hours. Then the excess of toluene and oxalyl chloride were removed by the mixture distillation (30ml). Afterward, filtration of the solvent from precipitate of sodium chloride performed under reduced pressure and dried in vacuum overnight to give anthraquinone-2-sulfochloride solid residue (4.36 grams, 90 % yield).


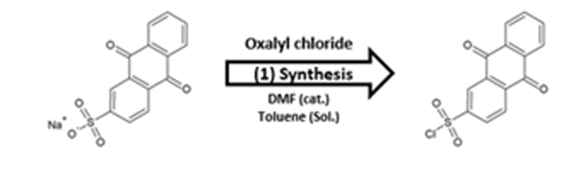


**Figure S10**. Sodium 9,10-anthraquinone-2-sulfonate conversion to 9,10 anthraquinone-2-sulfochloride

**8. Surface Modification of SiNW-FET Array with 9,10-anthraquinone-2 sulfochloride**

Bonding of 9,10-anthraquinone-2-sulfochloride to the SiNWs is possible as a result of the prior coupling of a monolayer of self-assembled APDMES to the surface of the sensor device. Before chemical modification, the sensor was cleaned with acetone, isopropyl alcohol (IPA), and DIW followed by nitrogen-stream drying and oxygen-plasma treatment. The sensor device was then inserted into a glove box (150B-G, Mbraun) under argon atmosphere and modified with (3-aminopropyl)-dimethyl-ethoxysilane to provide terminal amino groups on the nanowire surface. First, the chip was covered with ~150μl of APDMES (SIA0603.0, Gelest) for one hour, followed by a thorough rinse with anhydrous toluene (244511, Sigma- Aldrich). The chip was then removed from the glove box, washed again with IPA, dried under a gentle stream of nitrogen and baked at 115ᵒC for 30 minutes. Finally, a mixture of 50mg of 9,10-anthraquinone-2-sulfochloride, 20ml of anhydrous toluene and 1ml of anhydrous pyridine (270970, Sigma-Aldrich) was prepared in the glove box under argon atmosphere and the chip was immersed in this mixture overnight. This process resulted in a sulfonamide bond which connects the 9,10-anthraquinone group to the modified surface of the SiNW.


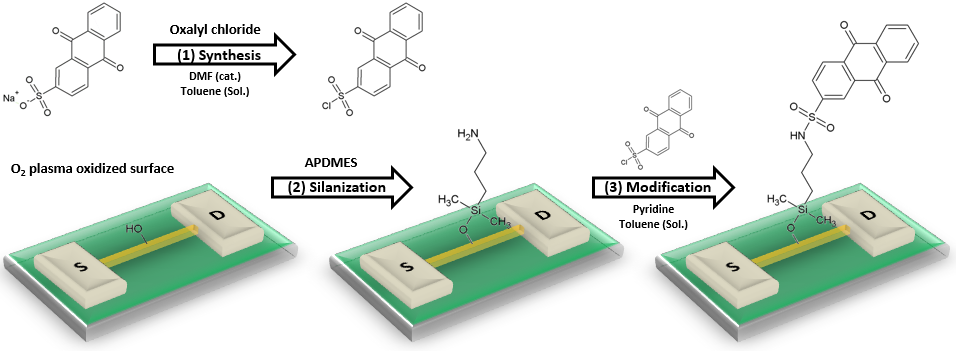


**Figure S11.** The modification procedure: (1) Sodium 9,10-anthraquinone-2-sulfonate conversion to 9,10-anthraquinone-2-sulfochloride; (2) Silanization of the activated surface of the SiNW with amine groups; (3) formation of a sulfonamide bond that connects the 9,10-anthraquinone to the modified surface.

**9. Fabrication of the MicroFluidic Channel as a Delivery System**

The fluid-delivery system was fabricated according to the SU8 template, from flexible polydimethylsiloxane (PDMS) elastomer, mixed in a 10:1 ratio with a base as a curing agent. The PDMS was cured overnight in an oven at 60ᵒC and then cut into rectangular pieces. The dimensions of the PDMS were 10×10×5 mm **(Figure S10).**

**
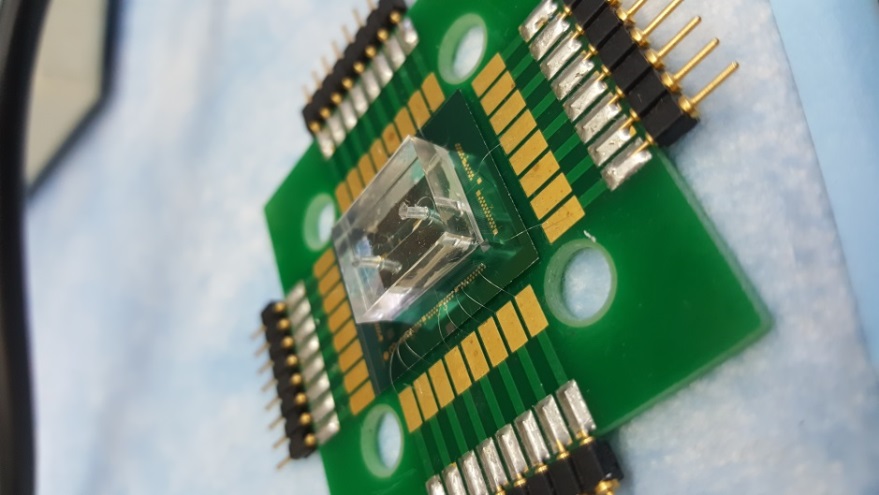
**

**Figure S12**. Image of a sensor chip clamped with PDMS microfluidic channel for delivery of solutions.

**10. Electrical Measurements System of SiNW-FET Devices**

The sensing system is assembled and connected to a computer in order to perform electrical measurements (**Figure S11**). The previously selected functional devices of the sensor chip are bonded to a printed circuit board (PCB) with wire bonding (using wire-bonder, model 8850, West Bond). Then, the PDMS microfluidic channel is mounted on the chip to be used as an active delivery system of fluid on the surface of the SiNW. A data-acquisition and signal-amplification digital system are connected to the PCB to enable the current recording of the SiNW-FET devices (Isd), with the use of a computer. Solution samples were injected by a syringe pump (Fusion 200, Chemyx), and the current was recorded at one-second intervals resulting in the conductance of the SiNW devices over time.

**
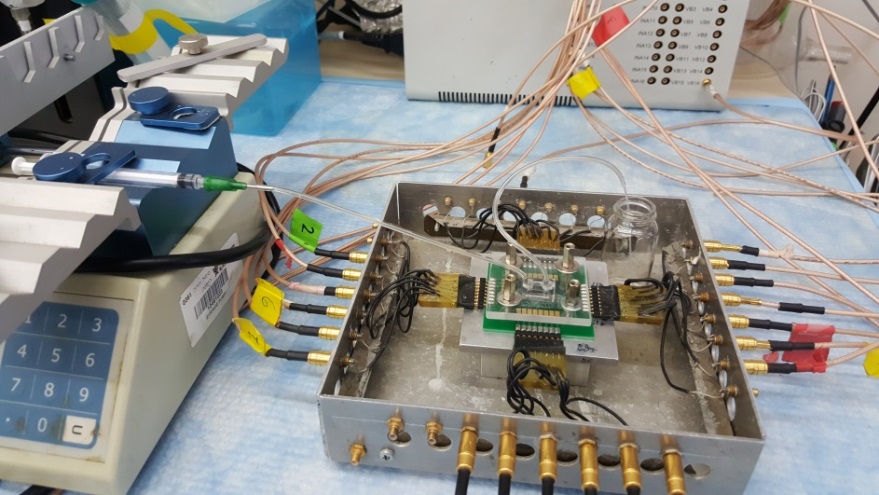
**

**Syringe Pump**

**Recording System**

**PCB holder**

**Figure S13**. The sensing system including the SiNW chip bonded to the PCB holder that is connected to the recording system. The microfluidic channel is placed directly above the SiNW array of the chip and attached via a clear perspex sheet and screws.

**11. E. coli Culture Handling**

Minimal broth medium: 7 g/L dipotassium phosphate, 2 g/L monopotassium phosphate, 1 g/L glucose, 1 g/L ammonium sulfate, 0.1 g/L magnesium sulfate, and 35 µg/mL chloramphenicol (diluted from a stock solution of 17.5 mg/mL in ethanol) at pH=7.3. All reagents were purchased from Sigma-Aldrich.

Starter of E. coli was taken from frozen E. coli stock (BL21(DE3)pLysS Singles™ Competent Cells, Merck Millipore) in 10 mL of 20 g/mL LB broth (Lennox, Acumedia), pH=7.5, and grown for 18 hours in 50 mL tube at 37 ºC by shaking at 250 rpm. Turbidity was followed using Ultrospec 10 cell density meter, Classic. When reaching 0.91 OD (compared to bacteria free minimal broth), 1 mL of starter solution was diluted in 100 mL of minimal broth medium at 4 ºC, stored in 1000 mL Erlenmeyer flask, and transferred to 37 ºC and 250 rpm shaking for bacteria growth and glucose consumption analysis. Prior to glucose metabolism detection, 20 µL GOX solution of 5mg/mL concentration were added to 1 mL of the E. coli in broth suspension.

**12. Formation and Maintenance of Bacterial Biofilms**

Growing of bacteria, and the process of forming bacterial biofilms, begin with the seeding of the bacterial colony from a frozen (-80ᵒC) stock of *B. subtilis* NCIB3610 wild-type strain (from Bacillus Genetic Stock Center), which expresses a yellow fluorescent protein (YFP) gene and kanamycin-resistance gene. The frozen bacteria are plated on an LB agar plate and incubated overnight at 37°C. One colony is taken with a loop and dipped into a test tube containing 2ml of LB medium, pH 7.5. The test tube is shaken at 37°C at 300 RPM to $OD_{600}$ 1.0 (starter culture). 25μl of the starter culture together with 25ml of MSgg medium is incubated at 30°C for at least 40 hours to form a pellicle (**Figure S12**). A pellicle is a biofilm that forms at the air-liquid interface and is usually used for the cultivation of microorganisms in microbiology. (LB broth and Agar Lennox, purchased from Acumedia).


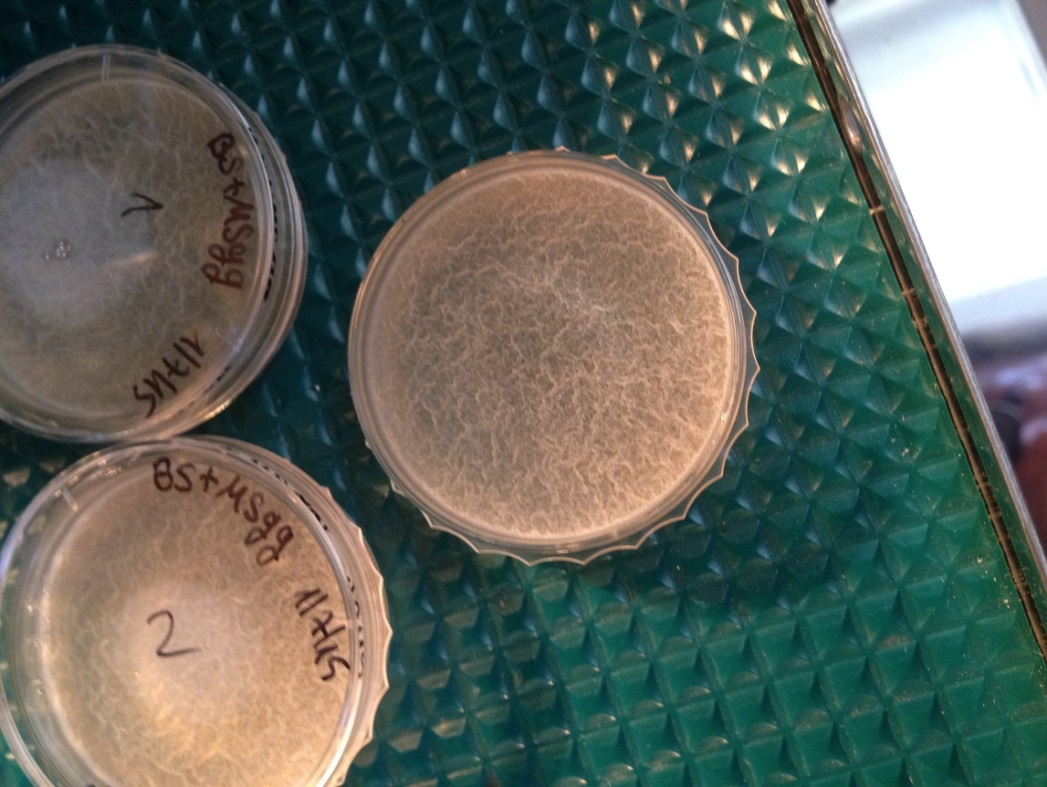


**Figure S14**. *B. subtilis NCIB3610* bacterial biofilm formed at the liquid-air interface in a petri dish after incubation of 40 hours in MSgg medium.

**Minimal medium MSgg** for bacterial growth: 5mM potassium phosphate pH 7, 100mM MOPS buffer pH 7 (1132612, Sigma-Aldrich), 2mM MgCl_2_ (7791186, Sigma-Aldrich), 700μM CaCl_2_ (10035048, Sigma-Aldrich), 50μM MnCl_2_ (13446349, Sigma-Aldrich), 50μM FeCl_3_ (7705080, Sigma-Aldrich), 1μM ZnCl_2_ (7646857, Sigma-Aldrich), 2μM thiamine, 0.5% glycerol, 0.5% glutamate, 50μg/ml tryptophan, 50μg/ml phenylalanine (All reagents were purchased from Sigma-Aldrich).

**Minimal broth medium** pH 7.3 containing glucose as the only carbon source was used as a sensing medium and maintenance medium after the formation of the biofilm in MSgg. The medium was composed of 7g/L dipotassium phosphate, 2g/L monopotassium phosphate, 1g/L d-glucose (without citrate), 1g/L ammonium sulfate and 0.1g/L magnesium sulfate. (All reagents were purchased from Sigma-Aldrich)

**1% v/v DEHA**: 1% by volume of N,N-diethylhydroxylamine (DEHA) (3710847, Sigma-Aldrich) in phosphate-buffered saline 150mM, pH 7.45 (tablet, Sigma-Aldrich).

**13. Measurement Protocol**

After the biofilm formed, the MSgg growth medium was withdrawn with a pipet and replaced with minimal broth medium (sensing medium). Immediately after replacement of the medium, sensing experiments begun with a known glucose concentration. A fraction of the bacterial medium was periodically collected and filtered (0.22μm). This fraction was divided in two, with each half containing 1ml of the solution, one untreated and the second mixed with 20μl of glucose oxidase (GOX) and incubated for 10 minutes. Sensing was performed on a SiNW-FET devices chip modified with a reactive redox layer, therefore reduction of the FET device was required before the measurements. Every injection of solution lasted three minutes; the source-drain voltage (Vsd) was set at 0.3V and the gate voltage (Vg) was set at 0V.

**14. Enzymes, Cofactors, Antibiotics, and Metabolites Used for Metabolic Analysis**

Glucose oxidase (GOX; G2133, Sigma-Aldrich), hydrogen peroxide ($H_{2}O_{2}$; 31642, Sigma-Aldrich), flavin adenine dinucleotide (F6625, Sigma-Aldrich), tetracycline (58346, Calbiochem), ampicillin (171254, Calbiochem).

**15. Error Analysis**

To estimate the error in the reported current values, the standard deviation (SD) of the current values 30 seconds before the devices were switched off (Vsd=0V), or the tubing were exchanged, was used. This estimation was performed in physiological solution of 1 vol% N,N-diethylhydroxylamine (DEHA), in order to avoid alterations in the current that are induced by the change in the oxidation state of the moieties bound to the surface of the redox-reactive FET device. The calculated SD was used for the estimation of the error in Y axis in **Figure 2b**.


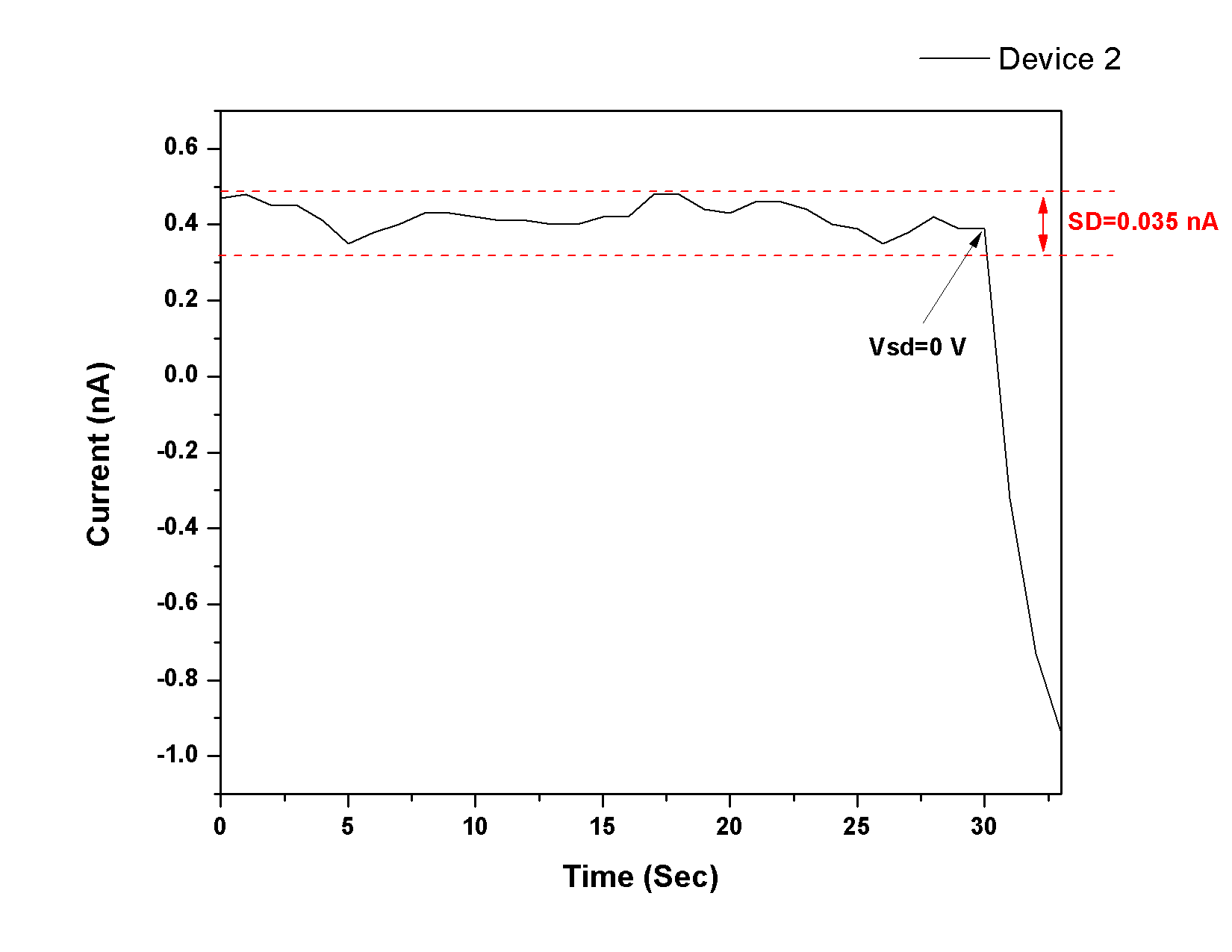


**Figure S15. Estimating the error in the current values (Y axis) in Figure 2b:** Calculation of the standard deviation (SD) of the current values, 30 seconds prior to switching-off the devices (Vsd=0V). The estimation was done in physiological solution of 1 vol% N,N-diethylhydroxylamine (DEHA). The sampling frequency of the current values was 1 Hz.

When the current values were calculated by subtracting one current from the other, the error was calculated according to the following equation:

$$when Z=A-B, the relation between the errors is \Delta Z=\sqrt{\left( \Delta A \right)^{2}+\left( \Delta B \right)^{2}}$$

The error in **Figure 2b** is estimated as described in the following equation:

$$\Delta Z=\sqrt{\left( SD \right)^{2}+\left( SD \right)^{2}}=\sqrt{\left( 0.07097 \right)^{2}+\left( 0.07097 \right)^{2}}=0.100\left[ \mathrm{nA} \right]$$

To estimate the error in the reported transmittance (T), calculated according to the measured absorbance (A) (**Figure 2a**), the following equation was used:

$$when T={10}^{-\left( A-A_{blank} \right)}, the relation between the errors is$$

$$\Delta T=2.023T\sqrt{\left( \Delta A \right)^{2}+\left( \Delta A_{blank} \right)^{2}}$$

**References**

1. Ishikawa FN, Curreli M, Chang H-K, Chen P-C, Zhang R, Cote RJ, Thompson ME, Zhou C: A Calibration Method for Nanowire Biosensors to Suppress Device-to-Device Variation. ACS Nano 2009; 3:3969-3976.

2. Gomes A, Fernandes E, Lima JLFC: Fluorescence probes used for detection of reactive oxygen species. J. Proteom 2005; 65:45-80.

3. Bergmeyer H: Methods of Enzymatic Analysis, I. Fundamentals. Wiley-VCH, Weinheim, Germany; 1983.

4. Patolsky F, Zheng G, Lieber CM: Fabrication of silicon nanowire devices for ultrasensitive, label-free, real-time detection of biological and chemical species. ‎Nat. Protoc. 2006; 1:1711.

5. Cui Y, Wei Q, Park H, Lieber CM: Nanowire nanosensors for highly sensitive and selective detection of biological and chemical species. Science 2001; 293:1289-1292.
